# Myelin ensheathment and drug responses of oligodendrocytes are modulated by stiffness of artificial axons

**DOI:** 10.1371/journal.pone.0290521

**Published:** 2025-01-24

**Authors:** Mingyu Yang, Calliope J. L. Martin, Kavin Kowsari, Anna Jagielska, Krystyn J. Van Vliet

**Affiliations:** 1 Harvard-MIT Health Sciences and Technology, Massachusetts Institute of Technology, Cambridge, Massachusetts, United States of America; 2 Department of Materials Science and Engineering, Massachusetts Institute of Technology, Cambridge, Massachusetts, United States of America; 3 Department of Mechanical Engineering, Massachusetts Institute of Technology, Cambridge, Massachusetts, United States of America; 4 Department of Biological Engineering, Massachusetts Institute of Technology, Cambridge, Massachusetts, United States of America; IRCCS Ospedale Casa Sollievo della Sofferenza: Ospedale Casa Sollievo della Sofferenza, ITALY

## Abstract

Myelination is a key biological process wherein glial cells such as oligodendrocytes wrap myelin around neuronal axons, forming an insulative sheath that accelerates signal propagation down the axon. A major obstacle to understanding myelination is the challenge of visualizing and reproducibly quantifying this inherently three-dimensional process *in vitro*. To this end, we previously developed artificial axons (AAs), a biocompatible platform consisting of 3D-printed hydrogel-based axon mimics designed to more closely recapitulate the micrometer-scale diameter and sub-kilopascal mechanical stiffness of biological axons. First, we present our platform for fabricating AAs with tunable axon diameter, stiffness, and inter-axonal spacing. Second, we demonstrate that increasing the Young’s modulus *E* or stiffness of polymer comprising the AAs increases the extent of myelin ensheathment by rat oligodendrocytes. Third, we demonstrate that the responses of oligodendrocytes to pro-myelinating compounds are also dependent on axon stiffness, which can affect compounds efficacy and the relative ranking. These results reinforce the importance of studying myelination in mechanically representative environments, and highlight the importance of considering biophysical cues when conducting drug screening studies.

## Introduction

Myelin is a lipid-rich membrane that ensheaths neuronal axons, thereby accelerating the efficiency of electrical signal conduction down the length of the axon [1,2]. Myelin is critical for the homeostatic function of mammalian nervous systems. Oligodendrocytes produce myelin in the central nervous system (CNS) and are fundamental to CNS development and myelin regeneration following injury [3,4]. During development, oligodendrocytes derive from oligodendrocyte progenitor cells (OPCs), which are maintained throughout adulthood and account for approximately 2–8% of the cells in the adult CNS [5]. Oligodendrocytes and OPCs inhabit a dynamic microenvironment within the CNS, which provides both biochemical and biophysical cues that regulate oligodendrocyte function. A growing body of evidence demonstrates that oligodendrocytes and OPCs are sensitive to external mechanical cues [6] including ECM stiffness (and more broadly the stiffness of the material to which OPCs adhere) [7–11], mechanical strain [12], macromolecular crowding, and physical confinement [13,14].

The sensitivity of OPCs to biophysical cues may play important roles in myelin pathology and neurodegenerative diseases. For example, neuroimaging data from human patients show correlations between neurological disease states, such as progressive multiple sclerosis (MS), and changes in the structural integrity and mechanical properties of brain tissue [15,16]. These findings are supported by murine models of demyelination, in which the Young’s modulus *E* of brain parenchyma decreased in response to acute demyelination from 240 Pa to 120 Pa, signifying a stiffness reduction correlated with disease progression [17,18]. In addition to tissue stiffness, other biophysical cues such as axon diameter and spacing may play important roles in oligodendrocyte biology and pathology. Under healthy conditions, axon diameter in the CNS can vary in diameter from 0.1–5.0 µm [19,20]. Magnetic resonance imaging (MRI) data has shown axon swelling to be a major pathological feature in chronic MS [21], and a similar case of increased axonal diameter was reported for amyotrophic lateral sclerosis (ALS) [22]. Furthermore, axonal injury and loss of axon density (number of axons per unit area or volume) is a hallmark of progressive MS [23], especially for chronically demyelinated axons. An open question is whether these biophysical changes simply act as correlative biomarkers or contribute to disease progression. In other words, do changes in axon diameter and brain parenchyma stiffness occur as secondary byproducts of myelination pathology, or do they actually change the propensity of oligodendrocytes to myelinate?

One approach for exploring these questions is to recapitulate and control these cues in an *in vitro* model. Prior examples of cell-free physical models of axons include electrospun fibers and glassy polymers. Although these models can recapitulate the geometry of biological axons, they use materials with Young’s modulus in the megapascal and gigapascal range, several orders of magnitude stiffer than biological axons [14,24–29]. With such materials, it is not possible to explore how changes in Young’s moduli within the sub-kilopascal physiological range can modulate myelination; nanometer-scale diameters and spacing of those stiff fibers can also challenge optical image-based validation of myelin wrapping around the fibers. To that end, we have previously developed artificial axons (AAs), which are 3D-printed hydrogel structures that mimic the sub-kilopascal stiffness and micrometer-scale diameter of biological axons [30–32]. Importantly, in this platform we can engineer properties of the axon arrays, such as stiffness, spacing, and diameter, enabling systematic studies of the influence of each cue on myelin wrapping. OPCs seeded on the AAs differentiate into mature oligodendrocytes and ensheath the axons with myelin, which we quantify in 3D.

While the AAs reported previously were a demonstrated proof of concept, scalability was limited and three-dimensional (3D)-printing of AAs on individual coverslips required at least two weeks to create sufficient samples for testing (for instance, to fill a 96-well plate). Here we describe advancements in design and fabrication of the AA platform, in which we 3D-print AAs directly into a 96-well plate using a digital mask, producing AAs with independently tunable Young’s modulus, axon diameter, and inter-axonal spacing. With this new platform, we significantly reduced sample damage to increase yield and reduced the total fabrication time to two hours, enabling production of a 96-well plate in which each well could represent a distinct combination of axon stiffness, diameter of spacing. We leveraged this tunability to explore how each parameter affects myelin ensheathment by rat oligodendrocytes. Finally, we focused specifically on Young’s modulus *E* and investigated how axon stiffness influenced oligodendrocyte responses to pro-myelinating compounds. We found that the relative efficacy of different pro-myelinating compounds, as quantified by a myelin wrapping index, differed between the AAs of higher (*E* =  13 kPa) and lower stiffness (*E* =  0.80 kPa) axons, with different “top 3” drug response rankings identified in each condition. In summary, we demonstrate that the AA platform is amenable to quantifying correlative and discovering causal relationships between axon biophysical cues and myelin ensheathment by oligodendrocytes, and that the mechanical environment can influence oligodendrocytes’ response to soluble biochemical cues including pro-myelinating compounds.

## Materials and methods

### Ethics statement

This study was carried out in accordance with the guidelines of the National Institutes of Health for animal care and use (Guide for the Care and Use of Laboratory Animals) and the protocol was approved by the Institutional Animal Care and Use Committee at the Massachusetts Institute of Technology (MIT Committee on Animal Care).

### Fabrication of artificial axons

**Fig 1:**
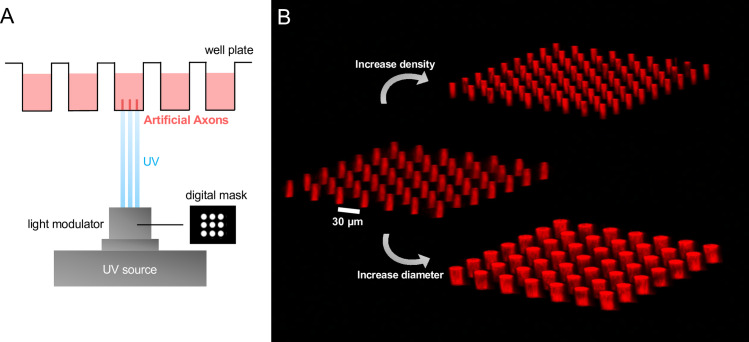
(A) Schematic of artificial axons fabrication using a digital photomask. (B) Confocal micrographs of printed artificial axons with tunable spacing and diameter.

Artificial axons (AAs) were fabricated directly in 96-well plates, using a projection microstereolithography setup. To vary axon stiffness (material Young’s modulus) the resins were prepared with varying ratios of 1,6-hexanediol diacrylate (HDDA) (Sigma-Aldrich) and 4-arm PEG acrylate (starPEG) (JenKem) monomers, at mass ratios of 3:1, 2:1, and 1:1. The resins were pipetted into the wells of 96-well plates and exposed to UV light using our custom-made projection microstereolithography setup [31]. Projecting the UV light onto the resin through the digital mask (Fig 1A) caused the liquid monomer resin to polymerize into solid vertical pillars that match the geometric pattern of the mask. The shape of the mask, the composition of the resin, the UV exposure duration, and the UV exposure intensity could all be modified to independently tune the Young’s modulus, diameter, and spacing of the axons (e.g., Fig 1B). For convenience, for the experiments on drug-induced myelination on different stiffness axons, we used a physical mask with the area corresponding to the area of the entire plate bottom, to generate axons in all plate wells simultaneously. Prior to introduction of rat oligodendrocyte progenitor cells, the AAs were functionalized with poly-D-ornithine (Sigma-Aldrich) (50 µg/mL) followed by incubation with laminin (Gibco) (20 µg/mL) to facilitate cell adhesion. The completed AA plates were stored in PBS at 4 °C and warmed to 37 °C the day of OPC seeding.

### Mechanical characterization of AAs

The Young’s modulus *E* of the cured AA material was determined using atomic force microscope (AFM)-enabled nanoindentation measurements (MFP-3D Bio, Asylum Research). Cylindrical structures of each material (10 µm thickness and width) were fabricated by projection microstereolithography using the same printing conditions as the AAs, and equilibrated overnight in PBS. AFM measurements were performed using a cantilever with nominal spring constant *k* =  0.1 N/m, terminating in a poly(methyl methacrylate) spherical probe with approximate diameter 1.5 µm (NanoAndMore). The actual spring constant was calibrated via the thermal noise method [33]. Between 30 and 40 force-depth responses were collected from each sample of the material. The cantilever base velocity was 1 µm/s, and probe retraction was triggered after reaching a maximum force of 30–100 nN, with lower forces for the more compliant samples. Young’s moduli *E* were calculated by fitting the spherical Hertzian elastic contact model [34] for data acquired up to an indentation depth of 200 nm.

### 3D myelin wrapping assay

Rat oligodendrocyte progenitor cells (rOPCs) were isolated from neonatal rat brains (postnatal day 1) using magnetic sorting with beads coated with A2B5 antibodies (Miltenyi). The isolated cells were expanded in tissue culture flasks for 2–3 days in proliferation medium containing DMEM/F-12 media (Gibco), penicillin-streptomycin (Gibco), B-27 (Gibco), 10 ng/mL each of platelet-derived growth factor (PDGF) (Gibco), and fibroblast growth factor (FGF) (Gibco). Expanded rOPCs were seeded in 96-well AA plates at a density of 20,000 cells per well in differentiation medium, consisting of DMEM/F-12 media, B-27, and 2 ng/mL each of PDGF and FGF. Only the inner 60 wells were used to avoid the outermost perimeter of wells which had accelerated liquid evaporation. Then 24 hours after seeding, 1/3 of the media was changed with fresh differentiation medium supplemented with a pro-myelinating drug. As the control condition, we used media containing 0.1% dimethyl sulfoxide (DMSO), which was the solvent vehicle used for other compounds. Media exchange of 33% volume occurred every other day, and cells grew on the AAs for either seven or 14 days before being fixed and stained. Every condition was repeated in at least triplicate, and two independent biological replicates (separate rounds of cell culture with two different rOPC batches) were conducted.

### Immunostaining

Cells were fixed with 4% paraformaldehyde (Electron Microscopy Sciences), washed three times with phosphate buffered saline (PBS), and permeabilized with 0.1% v/v Triton X-100 in PBS for 5 minutes at room temperature. Then, cells were washed three times in PBS and blocked for 1 hour in 5% v/v goat serum in PBS for 1 hour at room temperature. Cells were incubated in primary antibody (rat anti-MBP, BioRad, 1:200 dilution) for 24 hours at 4 °C. Next, cells were washed three times in PBS and incubated with secondary antibody (Alexa-Fluor-647 goat anti-rat, Thermo Fisher, 1:200 dilution) for 1 hour at room temperature. Cells were then washed three times in PBS and incubated with DAPI (Thermo Fisher, 1:1000 dilution) for 5 minutes. Finally, cells were washed once more and stored in PBS at 4 °C.

### Fluorescence imaging

Stained samples were imaged under three fluorescent channels (Alex-Fluor 647 for MBP + myelin, rhodamine for AAs, DAPI for nuclei) using a confocal microscope (Olympus, FluoView 3000) with 20x air lens. For the axon biophysical parameter experiments (for specific artificial axon stiffness, spacing, and diameter) in Fig 3, six to eight fields of view were imaged for each well, with an 8-slice confocal z-stack separated by a step size of 2 µm. Collectively, ~ 5000 axons per well imaged. For drug response experiments (Fig 4), nine fields of view were imaged with a 10-slice confocal z-stack separated by a step size of 2 µm. Collectively, ~ 10,000 AAs were imaged and analyzed per well.

**Fig 2:**
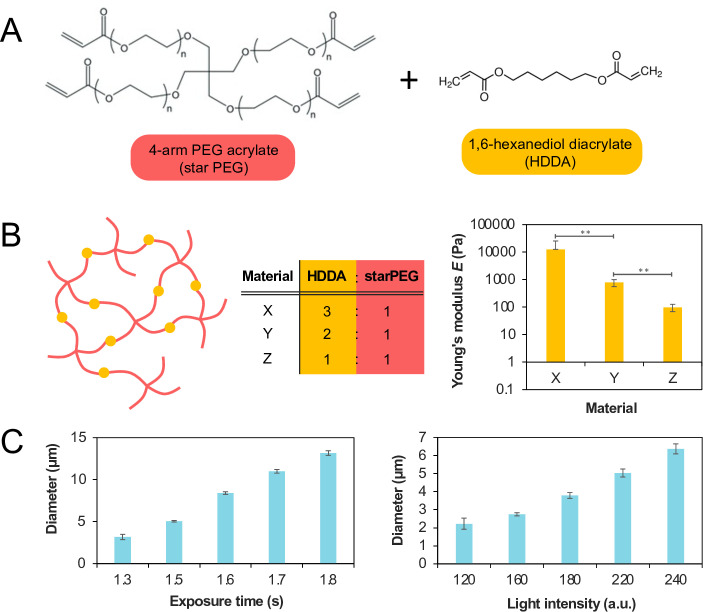
(A) Chemical structure of 4-arm PEG acrylate and 1,6-hexanediol diacrylate, the two main components of the polymer comprising these artificial axons. (B) Schematic of network structure formed by UV-mediated crosslinking of starPEG and HDDA. Materials X, Y, and Z are defined as having a 3:1, 2:1, and 1:1 HDDA:starPEG ratio respectively. Young’s elastic modulus (*E*) measured for three artificial axon samples with different HDDA:starPEG weight ratios. Error bars represent standard error of the mean. (C) Tunability of AA diameter by varying the UV exposure time and light intensity. Light intensity is measured on an arbitrary scale from 0 to 255. Data shown for Material X, and error bars represent standard error of the mean. In each condition, nine arrays of 15 × 15 axons were fabricated (2025 axons per condition). The diameters of all axons for a given condition were measured to obtain the mean and standard error values.

**Fig 3:**
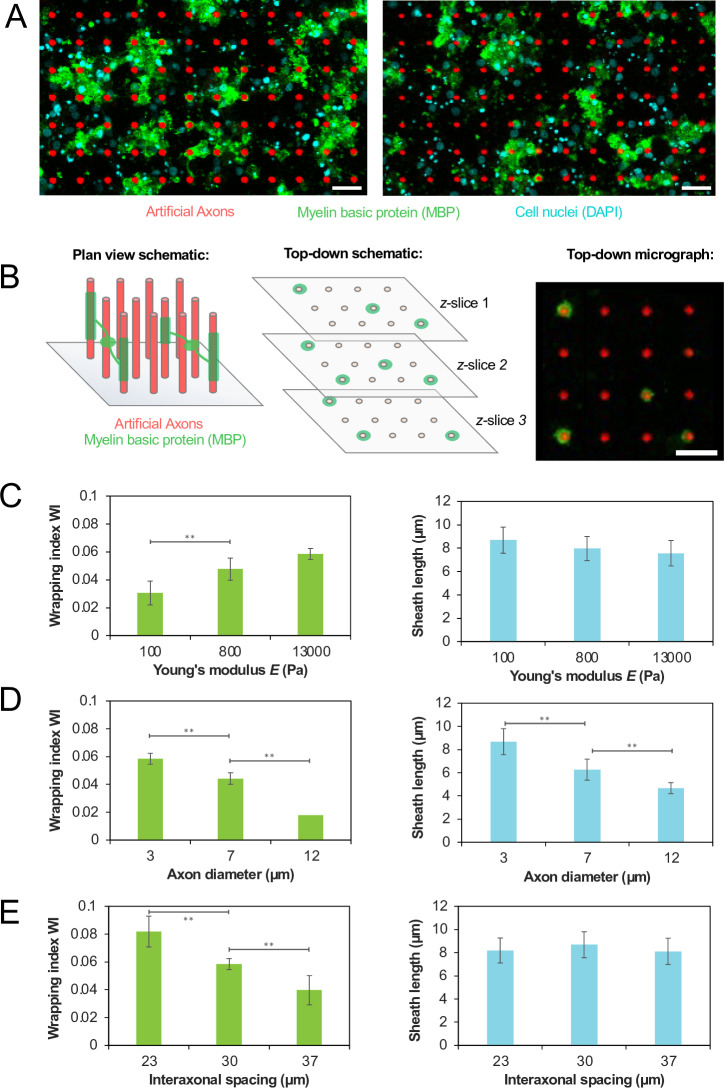
(A) Representative confocal micrographs showing AAs (red) wrapped with myelin basic protein (MBP, green) by oligodendrocytes (nuclei in blue), for stiffer axons made of material X (left panel, 13 kPa) and more compliant axons made of material Z (right panel, 0.1 kPa), indicating more wrapping of stiffer axons compared to axons with low stiffness characteristic of lesioned tissue. Scalebar in each micrograph is 30 µm. (B) Schematic of image analysis pipeline, in which 3D myelin wrapping is visualized by aggregating multiple *z*-slices from confocal microscopy. Scalebar in micrograph is 30 µm. The example image was intentionally selected to include a low number of wrapped artificial axons, and to not depict cell nuclei, for visual clarity. Variation of oligodendrocyte myelin wrapping with (C) Young’s modulus(D) diameter, and (E) density. The left graph shows wrapping index WI, a measure of the *number* of AAs wrapped. The right graph shows the distribution of myelin sheath lengths on AAs. In panels (C) and (D), inter-axonal spacing was held constant at 30 µm. In panels (C) and (E), axon diameter was held constant at 3 µm. In panels (D) and (E), Young’s modulus was held constant at 13 kPa. For each condition, there were three wells in replicate, each with 6–8 fields of view acquired, resulting in ~ 5000 axons imaged per well; all data were pooled and averaged across all n ≥  18 fields of view. A second independent experiment resulted in qualitatively the same trends, and those results are included in the S1 Fig.

**Fig 4:**
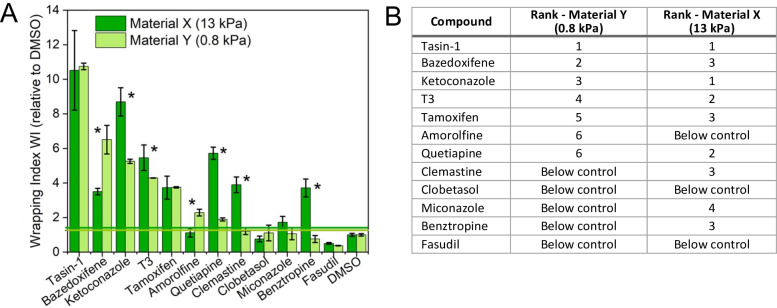
(A) Effect of stiffness on promyelinating activity of compounds. (A) Wrapping index WI for compounds scaled by control (WI for DMSO) on stiffer (Young’s modulus 13 kPa, dark green) and more compliant axons (Young’s modulus 0.8 kPa, light green). Error bars are standard error of the mean. (*) Statistically significant difference between responses on materials X and Y, p <  0.05. Horizontal lines denote 2 standard deviations above the respective DMSO controls. (B) Rank order of WI for compounds can differ on more compliant (0.8 kPa) and stiffer (13 kPa) axons. Materials X and Y are HDDA-starPEG hydrogels differing in extent of crosslinking resulting in different material stiffness. The results were obtained for drug concentrations corresponding to the maximum WI on material X (3 μM (ketoconazole, clemastine, benztropine, quetiapine, clobetasol, fasudil and miconazole) or 100 nM (tasin-1, tamoxifen, amorolfine, bazedoxifene, and T3). The graph represents average from two experiments. In each experiment, for each compound we used 3 well replicates with 9 fields of view per well; all data were pooled and averaged across all n =  27 fields of view. Comparison of the results from two independent experiments is included in the S2 Fig.

### Quantification of 3D myelin wrapping on AAs

The fluorescence optical *z*-slice images were processed through a custom image analysis pipeline. In brief, the AA, MBP + , and DAPI channels were imported to ImageJ and thresholded to obtain binary masks, followed by 3D volume reconstructions of the *z*-stacks. A 1-pixel-thick outline was traced around each AA; the outline was compared to the myelin mask to quantify the fraction of each axon circumference ensheathed by myelin. A 1-pixel-thick outline was traced around each AA; the outline was compared to the myelin mask to quantify the fraction of each axon circumference ensheathed by myelin. Aggregating across all *z*-slices, AA pillars were classified as “fully wrapped” if they exhibited a contiguous > 6 µm ensheathed segment length in which the AA pillar was > 80% wrapped in oligodendrocyte-synthesized myelin membrane positive for myelin basic protein (MBP) across all *z*-slices of that pillar height. For each field of view, a myelin wrapping index (WI) was calculated, as the number of fully wrapped artificial axons normalized by the number of cell nuclei.

### Statistical analysis

In Fig 2, for each condition, nine arrays of 15 × 15 axons were made, and the diameters were pooled and averaged across all axons of a given condition. In Fig 3, for each condition, there were 3 wells in replicate with 6–8 fields of view per well; all data were pooled and averaged across all fields of view (n ≥  18); collectively ~ 5000 artificial axons were analyzed per well. The experiment was repeated twice; the results of the second experiment are shown in S1 Fig. In Fig 4, the values represent average WI from two experimental repeats. In each experiment, for each condition there were 3 wells in replicate with 9 fields of view per well; collectively ~ 10,000 artificial axons were analyzed per well; all data were pooled and averaged across all fields of view (n =  27). Results from the individual experiments are shown in S2 Fig.

In Fig 2, for pairwise comparisons between conditions, Mann Whitney Wilcoxon tests were conducted using the SciPy package in Python. For three-way comparisons between three tested conditions, Kruskal-Wallis tests were performed using the SciPy package. In Figs 3 and 4, pairwise comparisons between conditions were done using one way ANOVA with Bonferroni correction using Origin Pro software.

## Results and discussion

### Additive manufacturing of artificial axons

When seeking to compare effects of biophysical parameters of the axon on oligodendrocyte wrapping potential, particularly in conjunction with responses to variable such as compounds to promote myelination, repeatability of those biophysical features and scalability to multi-well plates is advantageous. To that end, first we needed to adapt a 3D-printing approach to provide sufficient throughput Fig 1A demonstrates that new fabrication approach in which we polymerize artificial axons (AAs) directly into a multiwell plate using digital photomask, which consists of an array of white circles on a black background. This approach that uses UV light to polymerize micrometer-scale solid structures uses apreviously developed a custom poly(HDDA-*co*-starPEG) resin which is liquid when unpolymerized, but solidifies into a columnar geometry when exposed to ultraviolet or UV light [30–32]. Our previously reported approaches utilized individual 5 mm-diameter coverslips to fabricate AA arrays, which had limited scalability and required at least two weeks to create sufficient samples for testing in a full 96-well plate. Furthermore, the delicate sample handling requirements led to low yield of useful coverslips, with disproportionate damage of the samples with AAs of lower diameter (<10 μm) and lower stiffness (<1 kPa).

This approach reduced the total fabrication time of a 96-well plate of AA arrays from two weeks to two hours. That enables technical and biological replicates in assays with or without addition of compounds that may affect the oligodendrocyte wrapping response around a given axon feature in that array. Subsequent optical imaging is also facilitated by this standard assessment of multiwell plates. The digital mask modifies the UV light incident upon the resin, thus giving rise to vertical axons that match the pattern of the mask. Modification of the parameters of the digital mask via custom LabView code provides the flexibility to tune the AA geometry on a per-well basis by applying distinct masks and UV light exposure time and intensity. Fig 1A shows the potential to independently vary axon diameter and inter-axonal spacing using the digital photomask approach, simply by varying the size and spacing of white dots in the digital mask.

### Fine-tuned control of AA stiffness and diameter

The projection microstereolithography approach for manufacturing AAs provides multiple knobs including the monomer chemistry and UV exposure conditions, for tuning AA properties. The two primary components of this AA material are illustrated in Fig 2A: 4-arm PEG acrylate (starPEG) and 1,6-hexanediol diacrylate (HDDA). Copolymerization of starPEG and HDDA forms a network polymer illustrated in Fig 2B. We combined HDDA and starPEG at three different mass ratios of 3:1, 2:1, and 1:1 and determined their Young’s moduli *E* using atomic force microscope (AFM)-enabled indentation (see Materials and Methods). In order of descending HDDA:starPEG ratio, the *E* of the three materials were found to be 13000 Pa ±  64 Pa, 780 Pa ±  11 Pa, 98 Pa ±  5 Pa, respectively. This trend was consistent with expectations because HDDA functions as a crosslinker between the much larger starPEG molecules in the polymer network. Therefore, increasing the HDDA:starPEG ratio represents an increase in crosslinking density and therefore anticipates a stiffer polymerized AA material. Importantly, Materials Y and Z are consistent with the range of stiffnesses reported for neuronal axons and neural tissue [37].

To investigate the role of UV exposure time and intensity on AA diameter, we produced a digital photomask comprising an array of single-pixel white dots on a black background. We projected a UV beam of this mask to polymerize Material X (3:1 HDDA:starPEG ratio), across a range of exposure times (0.5 to 2 seconds) and light intensities (100–250, measured on an arbitrary scale of 0–255). Finally, we used confocal microscopy and ImageJ [35] to quantify the distribution of resultant AA diameters. As expected, increasing both the UV exposure time and light intensity produced a concomitant increase in axon diameter. This is consistent with the free-radical mediated polymerization mechanism through which the AAs form. UV exposure initiates polymerization by imparting reactive free radicals onto the monomer species. Increasing the light intensity increases the quantity of free radicals produced, thereby causing more monomers to react and producing larger-diameter axons from the same 1-pixel digital mask. A similar argument holds for increasing the exposure duration. We observed that AA height was consistent across these printing conditions, at 15–18 µm. Furthermore, we found that UV exposure duration and intensity both had to exceed a critical threshold for polymerization (i.e., in order for any AAs to form). In sum, adjusting the monomer chemistry and exposure conditions enables fine control over both axon stiffness and diameter, thus paving the way to investigate how these parameters may influence myelination by oligodendrocytes.

### Influence of AA stiffness, diameter, and density on myelin wrapping by rat oligodendrocytes

Next we considered how specific physical and mechanical features of the axon may independently affect oligodendrocyte wrapping response. We fabricated AAs of three different magnitudes of stiffness, of diameter, and of spacing, with a given well representing change in one feature while maintaining the other two constant, and quantified the resulting myelin ensheathment by rat oligodendrocytes as a parameter we termed wrapping index, WI. Seeding OPCs isolated from neonatal rat brains on AAs within 96-well plates for 14 days with differentiation induced by T3 at concentration 1 µ M, we then fixed and stained for myelin basic protein (MBP), a marker of differentiated oligodendrocytes and also a major component of myelin itself. Fig 3A shows a schematic of our image analysis pipeline for quantifying 3D myelin ensheathment (see Materials and Methods). In brief, we used confocal microscopy to take multiple top-down *z*-slices of the myelin-ensheathed AAs, then used ImageJ to compare across *z*-slices to quantify myelin wrapping. A 1-pixel-thick outline was traced around each AA; the outline was compared to the myelin mask to quantify the fraction of each axon circumference ensheathed by myelin. Aggregating across all *z*-slices, we classified pillars were classified as fully wrapped if they had a contiguous > 6 µm ensheathed myelin segment in which the AA was > 80% wrapped around the AA pillar circumference across all *z*-slices. For each field of view, we calculated the wrapping index WI, defined as the number of fully wrapped axons normalized by the number of cell nuclei. This allowed us to compute a mean wrapping index for each AA stiffness, spacing, and density condition.

Importantly, we measured two attributes of three-dimensional myelin wrapping around these features. The first is the previously defined WI, which indicates the *number* of AAs wrapped. We also separately quantified the *length* of the imaged myelin sheaths along the AA, calculated by determining the number of adjacent *z*-stacks with > 80% wrapping. The data shown in Fig 3 represent one repeat of the experiment; the data for a second replicate repeat are shown in S1 Fig.

We observed that increasing axon stiffness correlated with increased WI (Fig 3B), meaning that on average more of the stiffer AAs were wrapped per OPC (left panel). Here, we held axon diameter and inter-axonal spacing constant at 3 µm and 30 µm, respectively. Average myelin sheath length decreased slightly with the increasing AA stiffness, although the differences between the individual tested conditions were not statistically significant (right panel). This raises the possibility that the decrease in stiffness local to OPCs or oligodendrocytes in demyelinating contexts [15,17] could possibly decrease the intrinsic propensity of oligodendrocytes to myelinate axons. Based on these data alone, this is still just a speculative hypothesis; furthermore, an important caveat is that in this experiment we vary stiffness on an *axon*, whereas previous data report on more global changes in stiffness of the brain *tissue* in demyelinating lesions, without capacity for delineating stiffness at the individual cell or axon level.

We further observed that increasing AA diameter correlated with decreased WI (Fig 3C). Here, we held axon Young’s modulus and inter-axonal spacing constant at 13 kPa and 30 µm, respectively. Furthermore, the myelin that *was* wrapped on the higher-diameter AAs exhibited shorter average lengths. We also verified that the lengths of the AAs were uniform across conditions (15–18 µm) and not contributing to these results. This observation is counter to that in other prior studies: data reported in literature for biological axons or for axon-mimicking (electrospun and much stiffer) fiber meshes with fiber diameters <  2 µm demonstrates that within that range the larger-diameter axons are preferentially myelinated [36]. In our present study artificial axon diameters ranged from 3 to 12 µm, which can model swollen axons in the inflammatory demyelinating lesions (and are also relevant to the axon diameters in the peripheral nervous system). For this higher range of diameters, we observed the opposite trend for myelin wrapping: WI decreased with increasing axon diameter. As obtaining small artificial axon diameters with sub-kilopascal stiffness is challenging, we continue to further refine of our platform to exhibit sub-2 µm axon diameter variation, closer to the biological range of axon diameters in the central nervous system (CNS).

Finally, we found that increasing the mean separation between axons (in other words, reducing the axon array’s spatial density), which can model decreased axon density in chronic lesions, also decreased the WI (Fig 3C) though we observed minimal effect on sheath length. In this experiment, we held axon Young’s modulus and diameter at 13 kPa and 33 µm, respectively. Thus, differences in AA stiffness, diameter, and spacing can all influence myelin wrapping by oligodendrocytes principally by affecting the *number* of AAs wrapped (captured by the WI), although axon stiffness and diameter can also influence the *lengths* of the myelin sheaths deposited on the AAs by maturing oligodendrocytes.

### Influence of AA stiffness on response to pro-myelinating compounds

Based on the observed influence of axon stiffness on myelin wrapping (Fig 3B), we hypothesized that the responses of oligodendrocytes to pro-myelinating compounds may also depend on axon stiffness. To test this hypothesis, we conducted myelin wrapping assay in the presence of several pro-myelinating compounds acting on various ligands and signaling pathways, on axons with two distinct stiffnesses of 13 kPa (material X) and 0.8 kPa (material Y), with the AAs of lower stiffness corresponding approximately to that of normal axons [37]. Rat oligodendrocytes where cultured for 7 days and dosed every other day with a compound at concentrations of 3 μM (ketoconazole, clemastine, benztropine, quetiapine, clobetasol, fasudil and miconazole) or 100 nM (tasin-1, tamoxifen, amorolfine, bazedoxifene, and T3). The chosen drug concentrations corresponded to their maximum efficacy (measured as WI) in our previously published dose-response experiments for artificial axons comprising material X [38]. On day 8, we fixed the cells and immunostained for MBP.

For many but not all tested compounds, the WI differed in magnitude on artificial axons with differing stiffness (Fig 4). We observed higher WI on the stiffer axons upon oligodendrocyte exposure to ketoconazole, T3, quetiapine, clemastine, benztropine, and miconazole. By contrast, responses for bazedoxifene and amorolfine were weaker (i.e., lower WI) on stiffer axons compared to those corresponding to physiological stiffness of axons. We did not observe statistically significant differences as a function of AA stiffness in responses to tasin-1, tamoxifen, as well as clobetasol and fasudil for which WI was low overall and did not exceed the levels for the DMSO negative control condition. Notably, oligodendrocyte responses to amorolfine included WI significantly exceeding the DMSO response for axons of physiological stiffness, but insignificant wrapping above the negative control on stiffer axons. Conversely, some compounds inducing significant wrapping activity on stiffer axons (clemastine, benztropine, miconazole) showed negligeable activity on more compliant axons at the same concentrations. Additionally, relative ranking of WI among the compounds (Fig 4B) differed when assessed on stiffer compared to more compliant axons (Fig 4B).

While the compound eliciting the highest WI under these dosages and conditions was the same for both AA stiffnesses (tasin-1), the compounds ranked second and lower varied and could only be distinguished statistically for the more compliant axons. This indicates that using assay with axons with significantly higher stiffness than the physiological values, could result in either over- or underestimating of compounds efficacy and some promising compounds could be missed. This supports the concept that evaluation of pro-myelinating potential should include assays with mechanical stiffness representative of the target environment. Several compounds that were reported in prior studies to have pro-myelinating activity *in vivo*, namely clemastine [25], benztropine [39] and miconazole [40], showed WI scores exceeding control conditions on stiffer axons, but not at the same concentration on more compliant axons. It is possible that concentrations of these compounds that were optimized on stiff axons were too low to show pro-myelinating activity on compliant axons. (Dose-dependent responses could be obtained in principle for each axon stiffness condition to test this hypothesis, but were beyond the scope of the present study that sought to consider whether changes in stiffness in isolation could affect relative oligodendrocyte response to compounds.) Clobetasol, which was shown to have pro-myelinating activity *in vivo* in mouse models of demyelination [40], did not show pro-myelinating activity in our assay in this experiment. It is possible that species differences (mouse vs. rat) or cell batch variations could contribute to this contrasting finding. For example, in our previous *in vitro* artificial axon study of rat OPC response to drug compounds we observed a significant effect of clobetasol in our assay; the cell preparation protocols of OPCs from neonatal brain tissue were the same as described in the present study, but necessarily the specific animal sources differed [38].

Interestingly, we observed high promyelinating activity of tasin-1 and ketoconazole in our assay, which is consistent with positive responses to those compounds in human organoid [41] and *in vivo* mouse studies [42]. Further, it is possible that the results obtained herein for neonatal oligodendrocytes may not fully reflect the behavior of adult oligodendrocytes. The intrinsic myelinating potentials of oligodendrocytes obtained from earlier and later stages of development can differ [43]; studies of adult-stage oligodendrocyte responses remain quite limited, with both technical challenges and ethical considerations. We note that the above results reflect specific combination of biophysical and biochemical environment (specific axon coating, axon stiffness range, concentrations of compounds, cell batch), and we do not claim generalizable results for all conditions or compounds. Rather, we consider this example a proof-of-concept of the importance of the mechanically correct environment for drug screening.

It is unknown whether the relative responses of oligodendrocytes to these compounds may be influenced by maximum available AA length or pillar height (18 μm), and this remains to be explored in the future work. We note that in this present study and in our earlier work [38], we chose that dimension to achieve an assay that balances two concerns: (i) pillar span long enough to observe initial stages of engagement and ensheathment by the cells; and yet (ii) pillar span short enough to enable rapid quantification for relative comparison of responses among compounds (observation on day 7, with data obtained via confocal 3D image scanning and high-volume, high-content image analysis). The goal in that approach was not to reflect physiological sheath lengths, but to facilitate early and rigorous comparison among conditions. We note that in one of our earlier studies we reported that if artificial axon span length is 100s of micrometers, the sheath length observed *in vitro* in this assay can approach sheath length distributions for rodent brain tissue [44].

## Summary

Understanding how biophysical cues influence the 3D process of axon engagement and wrapping, or myelin ensheathment, is aided by reductionist *in vitro* approaches that control each of those variables near physiological ranges, and to not just visualize but readily quantify that 3D response. Given the established mechanosensitivity of maturing oligodendrocytes, including in how they engage engineered mimics of neuronal axons, control of those features within physiological ranges also provides means to compare stronger drivers of that wrapping, either in the absence or presence of compounds that may stimulate that cell-cell response. In this study limited to rat oligodendrocytes, we developed AAs with tunable Young’s moduli or mechanical stiffness (from the Pa to the kPa range), diameters (3 to 15 µm) and inter-axonal spacing. Increasing the stiffness or spatial density of AAs corresponded in a concomitant increase in the mean *number* of AAs ensheathed by oligodendrocytes, as quantified by a myelin WI that was normalized for the total cell number. However, sheath segment lengths were minimally affected by such changes in stiffness or spacing of these artificial axons, which are fully cylindrical but still shorter overall length span than human neurons. Axon diameter can change during development or injury, and here within the studied diameter range (3 to 15 µm), both the number of AAs ensheathed and the mean segment lengths of that wrapping decreased within increasing diameter. Note that the previously reported data for oligodendrocytes engaging with the polystyrene fibers spanning the diameter range from 0.4 to 2 µm showed that within that range increasing axon diameter actually elicited greater myelin wrapping by oligodendrocytes [36]. These results reflect a drawback with the AA platform, which is that the investigated diameters are still slightly above the physiological range of the CNS. It is possible that within a smaller diameter range, we could instead observe a different response to that observed here. Although future work is needed to further improve the representation of physical cues of physiological axons (for instance, fabricating AAs with sub-3 µm diameter), these results demonstrate capacity to probe correlative and causal relationships between biophysical cues and myelin wrapping *in vitro*.

An important application of *in vitro* modeling is the potential to compare and identify drug compounds that could stimulate myelin repair in demyelinating diseases. We found that the relative efficacies of pro-myelinating compounds differed depending on AA stiffness, as quantified by the wrapping index, which may have implications for *in vitro* drug screening. For example, the compound candidates identified in the *in vitro* drug screens with oligodendrocytes cultured on high-stiffness substrata such as tissue-culture polystyrene (TCPS) or stiff nanofibers may not be representative of the 3D wrapping responses predicted in a more compliant biomechanical environment. As our results suggest, it is possible that drugs which *may* have been ranked highly in mechanically compliant environments could be missed when screening oligodendrocyte response in formats of superphysiological stiffness. Collectively, these results speak to the importance of studying myelination in mechanically representative environments. This flexible and reductionist artificial axons platform provides the ability to study individual biophysical cues that exist in the demyelinating lesions to understand how each of these factors may contribute to myelination and myelin repair, and how these biophysical factors can affect the responses to promyelinating compounds. Studies of such biophysical cues are not practically controllable in vivo and also challenging in organoids: there is an inherent challenge of separating different cues from each other and from multiple other factors in the animal’s CNS that influence myelination and drug response, also making mechanisms of drug action or failure challenging to discern. Combining these biophysical attributes of axon-like environments can aid rational understanding of glial cell-neuronal cell engagement, including features that may change with lesion or pathological state, and of pro-myelinating drug development.

## Supporting information

S1 Fig Variation of oligodendrocyte myelin wrapping with (A) Young’s modulus *E*, (B) diameter, and (C) density – Results from the second independent experiment. The left graph shows wrapping index WI, a measure of the *number* of AAs wrapped. The right graph shows the distribution of myelin sheath lengths on AAs. The data for panels B and C are for axons of *E* =  13 kPa axons. Error bars represent standard error of the mean. For each condition, there were three (3) wells in replicate with six to eight fields of view per well; all data were pooled and averaged across all n ≥  18 fields of view.
(TIF)

S2 Fig Comparison of the wrapping index (WI) for the panel of compounds between two independent experiments, for (A) material X and (B) material Y. Wrapping index values for compounds are scaled by the wrapping index of the DMSO control for each experiment. There were no statistically significant differences between relative responses of oligodendrocytes to compounds between the experiments (tested by one way ANOVA with Bonferroni correction). Error bars represent standard error of the mean. In each experiment, for each compound there were three (3) well replicates with nine (9) fields of view per well; all data were pooled and averaged across all n =  27 fields of view.
(TIF)
