# Supplementary figures and images for: Myelin ensheathment and drug responses of oligodendrocytes are modulated by stiffness of artificial axons
Source: PLoS One. 2025 Jan 24;20(1):e0290521. doi: 10.1371/journal.pone.0290521 (PMC11759361; doi:10.1371/journal.pone.0290521)

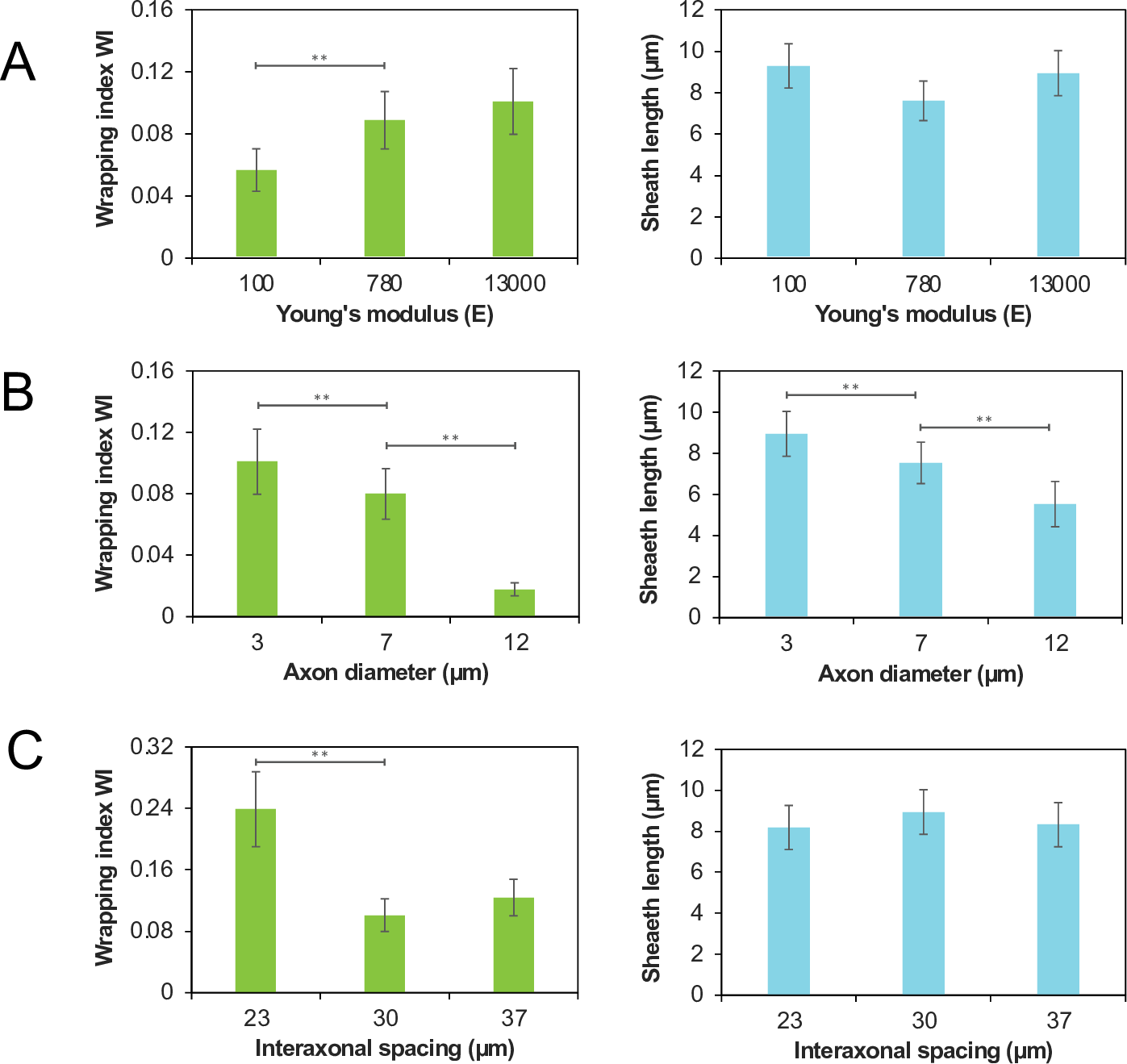

Supplement: S1 Fig — The left graph shows wrapping index WI, a measure of the number of AAs wrapped. The right graph shows the distribution of myelin sheath lengths on AAs. The data for panels B and C are for axons of E = 13 kPa axons. Error bars represent standard error of the mean. For each condition, there were three (3) wells in replicate with six to eight fields of view per well; all data were pooled and averaged across all n ≥ 18 fields of view. (TIF) [file pone.0290521.s001.tif]

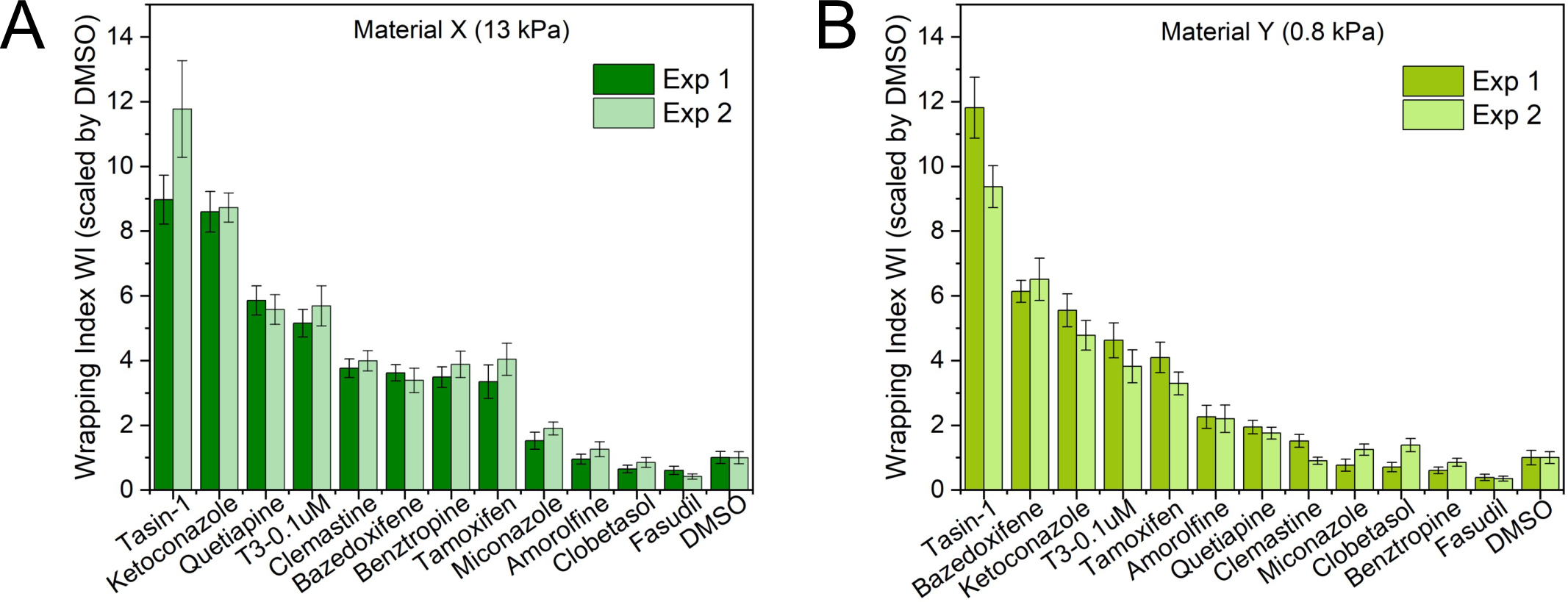

Supplement: S2 Fig — Wrapping index values for compounds are scaled by the wrapping index of the DMSO control for each experiment. There were no statistically significant differences between relative responses of oligodendrocytes to compounds between the experiments (tested by one way ANOVA with Bonferroni correction). Error bars represent standard error of the mean. In each experiment, for each compound there were three (3) well replicates with nine (9) fields of view per well; all data were pooled and averaged across all n = 27 fields of view. (TIF) [file pone.0290521.s002.tif]
